# Digital technologies for behavioral change in sustainability domains: a systematic mapping review

**DOI:** 10.3389/fpsyg.2023.1234349

**Published:** 2024-01-03

**Authors:** Oriana Mosca, Andrea Manunza, Sara Manca, Giuliano Vivanet, Ferdinando Fornara

**Affiliations:** Department of Pedagogy, Psychology and Philosophy, University of Cagliari, Cagliari, Italy

**Keywords:** systematic mapping, digital technologies, sustainable behaviors, gamification, power-metering

## Abstract

Sustainability research has emerged as an interdisciplinary area of knowledge about how to achieve sustainable development, while political actions toward the goal are still in their infancy. A sustainable world is mirrored by a healthy environment in which humans can live without jeopardizing the survival of future generations. The main aim of this contribution was to carry out a systematic mapping (SM) of the applications of digital technologies in promoting environmental sustainability. From a rigorous search of different databases, a set of more than 1000 studies was initially retrieved and then, following screening criteria based on the ROSES (RepOrting standards for Systematic Evidence Syntheses) procedure, a total of *N* = 37 studies that met the eligibility criteria were selected. The studies were coded according to different descriptive variables, such as digital technology used for the intervention, type of sustainable behavior promoted, research design, and population for whom the intervention was applied. Results showed the emergence of three main clusters of Digital Technologies (i.e., virtual/immersive/augmented reality, gamification, and power-metering systems) and two main Sustainable Behaviors (SBs) (i.e., energy and water-saving, and pollution reduction). The need for a clearer knowledge of which digital interventions work and the reasons why they work (or do not work) does not emerge from the outcomes of this set of studies. Future studies on digital interventions should better detail intervention design characteristics, alongside the reasons underlying design choices, both behaviourally and technologically. This should increase the likelihood of the successful adoption of digital interventions promoting behavioral changes in a more sustainable direction.

## 1 Introduction

Digitalization and sustainability (or sustainable development) are key issues for policy-makers and practitioners in the 21st century (Di Fabio, 2021). In this regard, since the use of digital technologies has become widespread, a full comprehension of their role in sustainable development is desirable. Recently, sustainability science has been characterized as an interdisciplinary research area (Rosen, 2017), from the natural to the applied sciences, moving from engineering to social sciences and humanities. This interdisciplinary approach allows a comprehensive understanding of the complex interplay between the planet we live on and human well-being, by taking into account the intertwined relationships among humans, the environment, and engineered systems (Di Fabio, 2021). Indeed, the knowledge about how to reach sustainable development has grown, while policy development toward the goal is still in its infancy.

Research on sustainability is based on the concept of a balanced and harmonized life, at different levels, starting from the micro to the macro: individual, group and organizations and relationships among them, community, national, and trans-national levels (Di Fabio, 2021).

In 1987, the Brundtland Report, where the concept of sustainable development was first introduced, was published by the World Commission on Environment and Development (WCED), with a focus on possible answers to some critical threats and challenges which are still not solved nowadays, such as reaching an harmonization between economic development and environment protection, pollution reduction, the regulation of the exploitation of natural resources, harmful gas emissions, and climate impacts management, attainment of global peace, and hunger and poverty levels lowering. Sustainable development is defined as “development that meets the needs of the present without compromising the ability of future generations to meet their own needs” (WCED, 1987, p. 43), which became a widespread accepted and probably the most quoted definition in literature, commonly known as the “Brundtland definition”. Traditionally, sustainability is commonly described from a triple-bottom-line perspective, with a metaphor, that is the search for “balance” between three components, i.e., environment, economy, and society (the so-called “three-pillar model,” Hilty and Aebischer, 2015). Sustainability represents the act of balancing the pace of human development with the resources required to achieve such a goal. Indeed, in the XVIII century, Carl von Clausewitz stated that humans should not cut down trees at a rate faster than that at which they are replaced (Akande et al., 2019).

The economic area focuses on financial and economic outcomes, the social one is devoted to addressing inequalities and ensuring inclusion and accessibility of services and resources, while, the environmental area is related to protecting the environment from excessive pollution, like carbon emissions (Mensah, 2019).

Sustainability science has become closely interlaced with the spread of digitalization and more relevance has been put to digital technologies as instruments for improving global well-being (United Nations, 2015). Indeed, the UN has been working on an international agenda for sustainable development since 2016 and pointed at technology as an irreplaceable driver for reaching specific sustainable goals by 2030 (Camodeca and Almici, 2021). Sustainability and digitalization originated massive research about how these two fields of study, separately, changed the whole society (Camodeca and Almici, 2021). The intersection of these two domains, especially in the research of which digital intervention is the most effective to promote sustainability, however, remains a largely unexplored territory, with a few exceptions. For instance, Akande et al. (2019) focused on the role that Information and Communication Technology (ICT) has on carbon footprint decrease, which in turn fosters the sustainability of smart cities. In this study, ICT is an indicator of smartness, and CO_2_ emissions are used as an indicator of sustainability in different European cities, thus underlining the need for a common strategy for achieving integrated, smart, sustainable, and inclusive cities (Akande et al., 2019). Following a multi-method approach, Brenner and Hartl (2021) examined how different actors express the relationship between digitalization and sustainability in media discourses and which dimension of sustainability is predominant. The Authors found that the evaluation of ecological and economic sustainability (but not social) is influenced by the extent of digitalization. These findings call for a more nuanced view of sustainability that represents better its multifaced dimensionality, with a particular focus on the social dimension, to be promoted by multiple actors, like policy-makers and interested stakeholders.

Starting from the awareness of the scarcity of studies mapping digital transition in the environmental sustainability domain, Feroz et al. (2021) conducted a systematic literature review on the disruptions driven by digital transformations, which were organized in four key areas: pollution control, waste management, sustainable production, and urban sustainability. This mapping allowed to propose an agenda for future research in terms of digital transformation strategies, organizational capabilities, and performance in the field of environmental sustainability (Feroz et al., 2021). Examples of these two areas, digitalization, and sustainability, applied in the field of organizations, proliferate, starting from green technologies and production operations to transforming a classic company into a sustainable one. While a considerable amount of attention has been put on these shifts individually, less attention has been devoted to understanding how these trends can be combined. In fact, the possible exchange between these scientific research areas has been recently addressed, as witnessed by a growing number of studies based on the use of digital interventions to change people’s behavior in a more sustainable way (Hedin et al., 2019). These behavior change interventions used various techniques, such as gamification, nudging, and persuasive apps, just to mention a few of them (e.g., Comber et al., 2013; Hedin et al., 2019). However, despite the growing number of research studies in this intersection area, there is a clear lack of synthesized knowledge on the types of successful interventions behavior toward sustainable behaviors (SBs).

To fill such a gap, we present a systematic mapping review of the digital technologies used to promote sustainable behaviors, in order to trace trajectories of research in this area and to orientate both future research agenda and policy decision-making. To this end, the review includes an in-depth analysis of 37 selected articles, with a classification scheme (see Supplementary Table 1) showing multiple aspects covered in existing research. From our literature search, it emerged that systematic reviews on the relationship between digitalization and sustainability science have been conducted only for narrowed topics. For example, Hedin et al. (2019) published a systematic review on digital behavior change interventions in the field of sustainable food consumption. Another example of focused topics is the one by Castro et al. (2021), who recently published a holistic review about the confluence of digitalization and sustainability in promoting the Sustainable Development Goals. Specific contexts were considered, like the recent systematic review on the influence of environmental sustainability and digitalization processes on the business development of Small and Medium-sized Enterprises, SMEs (Isensee et al., 2020). It is worth noting that a recent mapping study has been published on the general and active use of only one category of digital technologies, i.e., serious games and gamification, to promote sustainable behaviors (De Salas et al., 2022). To create successful techniques to encourage environmentally friendly behavior, it is crucial to comprehend the psychological elements that underlie sustainable behavior. Indeed, intention to act is a strong indicator of action, according to psychological theories like Ajzen (1991), and knowing the variables that affect intention can help in designing interventions that will effectively encourage sustainable habits. Additionally, habits and social norms have a big impact on behavior, thus it’s necessary to comprehend these psychological aspects before designing interventions (Piras et al., 2021; Dessi et al., 2022, Bonaiuto et al., 2023). Different key theories informing sustainable technology adoption in a pro-environmental direction are available as potential frameworks to understand the attitude-behavior gap. These include the Diffusion of Innovations Theory (Rogers, 2010), the Technology Acceptance Model (Davis, 1989), and the Value-Belief-Norm Theory (Stern, 2000). Some typologies of digital technologies, like smart grids or virtual reality apps and games, can be considered as early-stage innovations. According to the Diffusion of Innovations Theory (Rogers, 2010), innovations are adopted through different stages, defining so five categories of adopters (i.e., innovators, early adopters, early-users, early majority, laggards) influenced by the characteristics of the innovation itself, communication channels, time, and the existing social context in which the innovation is developed. Another model that can be used as a useful framework connecting the psychology of sustainable behaviors and the use of digital technologies is the Technology Acceptance Model (Davis, 1989), which describes perceived usefulness and perceived ease of use as two key determinants of the adoption of various technologies. Different theories and models have been developed to explain several types of SBs. A review of these theories is outside the aim of this paper, however, one of the most used theories is the Value-Belief-Norm Theory (Stern, 2000). According to this theory, behavior is explained by a chain of sequential variables: biospheric and altruistic personal values predict ecological worldview, which gives rise to the awareness of consequences for the environment, that in turn increases the ascription of responsibility to the self for such consequences. Finally, this sequence triggers the sense of obligation to take action (i.e., the moral norm) which predicts the congruent pro-environmental behavior (Stern, 2000). Thus, personal values are foundational factors that influence SBs.

These theories and constructs could be helpful in shedding light on how to encourage environmentally responsible behavior and, eventually, contribute to a healthy world by approaching the issues of digital technology and sustainability from a psychological viewpoint. Indeed, digitalization could be viewed as a resource to close the gap between intention and action for sustainable lifestyles. Digital tools, like serious games, mobile apps, and smart meters, can have the potential to inspire sustainable behaviors and maintain them over time, stimulating personal engagement through the use of social norms and profiting, for example, from gamification approaches to support behavior change.

### 1.1 Objective and research questions

This contribution aims to systematically map the literature about digital behavior change interventions for actively promoting SBs, with the scope of improving the knowledge on the role that digital technologies can have in triggering individuals’ and groups’ sustainable actions. Specifically, this systematic mapping review is built to answer these research questions:

RQ 1 What are the main bibliometric characteristics of the selected articles?

This research question intends to find out the number of published articles in the selected period, the year of publication, and the places where the studies have been published.

RQ 1.1 What is the distribution of selected articles for years of publication?

RQ 1.2 What geographical distributions are being covered?

RQ 2 Which method features are represented in the selected articles?

This research question aims to analyze a set of method characteristics, including research types and approaches used, and areas targeted for data extraction.

RQ 2.1 Which research design was used to conduct the studies?

RQ 2.2 Which sampling technique was adopted?

RQ 2.3 What are the types of digital technologies and SBs in the selected studies? And what is the impact of the first ones on the second ones?

The main aim here is to detect the key facets of digital technologies (and their impact) involved in the promotion of sustainable behaviors.

RQ 3.1 What are the main digital technologies used to promote environmental sustainability?

RQ 3.2 What are the main SBs investigated?

RQ 3.3 How much evidence is available for long-term impacts - what is the actual length of studies?

RQ 3.4 For whom digital interventions are programmed?

RQ 3.5 What evidence is there concerning the efficacy of digital technologies interventions in promoting SBs?

## 2 Method

To provide an answer to the research questions, we carried out a systematic map (SM), a synthesis method whose aim is to collect, catalog, and describe the available evidence related to a broad topic (James et al., 2016), firstly conducted in the social sciences (Bates et al., 2007; Clapton et al., 2009) and then adapted for its use in environmental management and conservation science (Randall and James, 2012). SMs can be useful in providing reliable knowledge for researchers, decision-makers, and practitioners, for instance by helping to identify domains of knowledge requiring further research and/or reliable data suggesting best practices in a given domain (Haddaway et al., 2016). The ROSES (RepOrting standards for Systematic Evidence Syntheses; Haddaway et al., 2018) protocol, which is tailored to environmental systematic reviews and maps, consistently with Collaboration for Environmental Evidence (CEE) guidelines (Collaboration for Environmental Evidence, 2018; Haddaway et al., 2018), has been adopted to carry out the SM.

### 2.1 Search strategy

To retrieve primary studies focused on digital technologies’ use for promoting SBs, the following search strategy was adopted. Starting in June and ending in November 2020, we performed a comprehensive literature search in different databases (Web of Science, Scopus, PsycArticles, and ERIC). To this aim, the following combination of keywords related both to the intervention (use of different types of digital technologies) and the outcomes (SBs), was adopted and combined through Boolean operators: (“sustainable behavior” OR “pro-environmental behavior” OR “environmental behavior” OR “environmental psychology” OR “conservation behavior” OR “environmentally significant behavior” OR “environmentally supportive behavior” OR “ecological behavior”) AND (“technology” OR “simulation” OR “virtual reality” OR “digital games” OR “serious games” OR “artificial intelligence” OR “multimedia” OR “video” OR “software” OR “mobile” OR “information systems” OR “e-learning” OR “internet of things” OR “learning systems” OR “robot” OR “computer” OR “audio-visual” OR “virtual learning environment”).

### 2.2 Eligibility criteria

We adopted the following inclusion criteria for our SM review: (a) studies had to investigate the implementation of digital technologies interventions (Independent Variable) to promote SBs (Dependent Variable); (b) studies using different methods (i.e., qualitative, quantitative, or mixed methods) measuring the effect (experimental, lab-based, quasi-experimental, mixed methods, or field experiment) of digital technologies interventions through comparator (temporal - within-subjects - or experimental - between subjects - manipulation, mixed methods); (c) studies had to be published in the last 10 years (from January 2010 till November 2020) and had to be available in one of the following languages: English, Italian, Spanish, or French; (d) studies had to be published in academic and scholarly peer-reviewed journals. No constraints were planned for the reference populations. We also chose to exclude the following contributions: (a) work-in-progress studies, short papers, Ph.D. dissertations, and congress proceedings; (b) theoretical papers or cross-sectional and correlational studies; (c) digital interventions that have not been implemented (e.g., papers describing only the programming of a web app); (d) digital technologies only described but non used for an active intervention; (e) digital technologies not used as an antecedent of SBs; (f) interventions not been evaluated from a behavior change perspective; (g) interventions covering aspects other than the ones directly linked to SBs; (h) case studies without any inference; (i) model simulation studies; (j) studies with referenced links to other complementary evidence; (k) studies lacking fundamental information. Using the predefined eligibility criteria detailed above, papers were selected according to a three-stage hierarchical screening processing order to establish their eligibility: first, papers were evaluated considering language and documents’ type, subsequently by title, abstract, and keywords, and finally considering the full-text. Those papers for which there were doubts or insufficient information for the decision were retained for assessment at a later stage.

### 2.3 Categorization criteria

A concept-centric review was performed, on the basis of categories related to the type of digital technology employed for the promotion of different kinds of SBs. Research in the field was classified by using a coding scheme (Supplementary Table S1) in which the investigated dimensions (i.e., study characteristics like sampling strategies and research design; intervention characteristics like the employed digital technology and the promoted SBs; investigated outcome and population characteristics) are presented according to the ROSES framework.

## 3 Results and discussion

**Figure 1:**
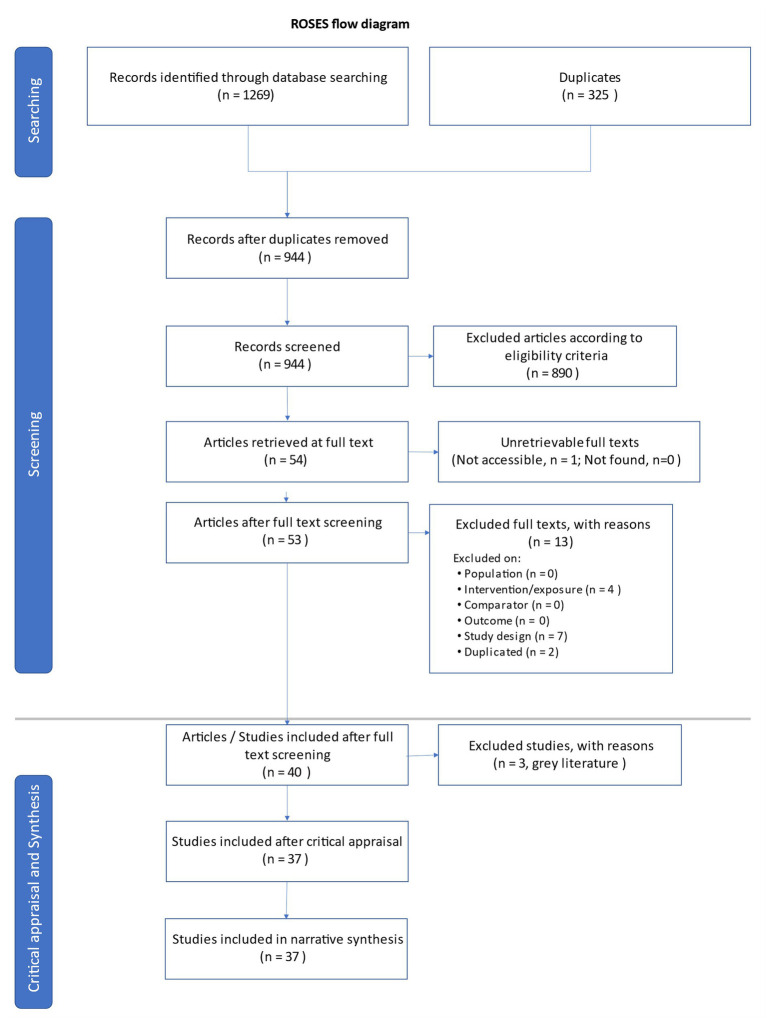
Flow-diagram illustrating articles/studies recovered in the initial search and included following screening and full-text assessment.

In total, 1.269 articles were retrieved (Figure 1). Duplicates (*n* = 325) were removed, and the articles screened for title, abstract, and keywords were 944. Consistently with the eligibility criteria, we excluded 890 of them, reaching a number of 53^1^ articles for the full-text screening. Subsequent analysis led to the exclusion of 4 studies for intervention-related reasons (i.e., technology not used as dependent variable), 7 studies for design-related reasons (i.e., a technology used as a mere instrument, with no empirical study), and 2 studies because they were duplicates but published with different versions. In sum, 40 articles were included after the full-text screening. Finally, 3 articles were removed because they belonged to gray literature (2 conference proceedings and 1 Ph.D. dissertation), and thus a definitive total of 37 studies were included in the review.

### 3.1 RQ 1: bibliometric characteristics of the selected articles

**Figure 2:**
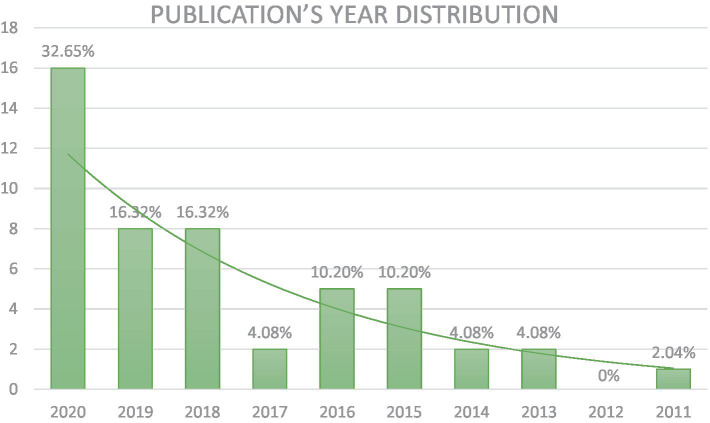
Publication’s year distribution of the screened articles.

The recency and the increase of scientific interest in this topic are demonstrated by the distribution of the selected papers by year of publication, presented in Figure 2.

In the considered publication time (2011–2020) there is a year-after-year after year increase in the number of published studies, with one occasional slowdown occurring in 2017.

**Figure 3:**
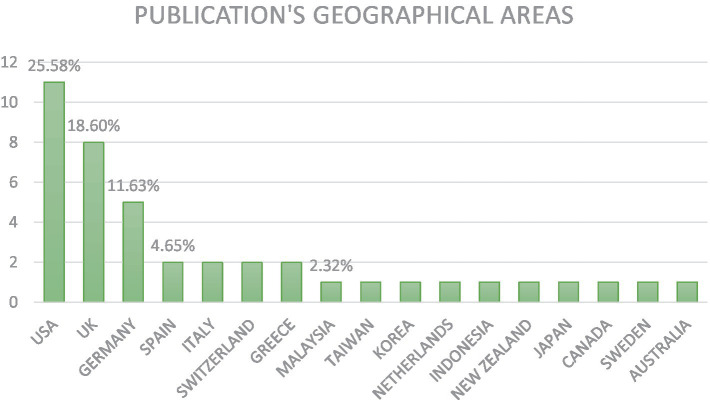
Geographical distribution of the screened articles.

Regarding geographical distribution, the following picture emerged (see Figure 3).

Studies from a total of 43^2^ countries were found with most studies originating in North America (e.g., United States of America (USA) – 25.58%, Canada 2.32%), United Kingdom (UK): 18.60%, and Europe (e.g., Germany: 11.63%, Spain: 4.65%, Italy: 4.65%, Switzerland 4.65%, Greece 4.65%, Netherlands 2.32%, Sweden 2.32%), few from Asia (e.g., Malaysia: 2.32%, Taiwan: 2.32%, Korea: 2.32%, Indonesia: 2.32%, Japan: 2.32%), and few from Oceania (Australia: 2.32%, New Zealand: 2.32%), in some respects, reflecting the geographic spread of OECD countries around the globe, except for the Asian countries.

### 3.2 RQ 2 research type facets

**Figure 4:**
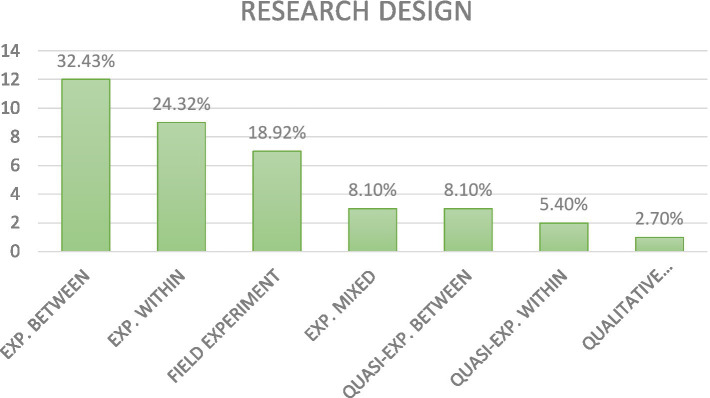
Research design used in the screened articles.

Regarding the kind of research design, the following picture emerged (Figure 4).

**Figure 5:**
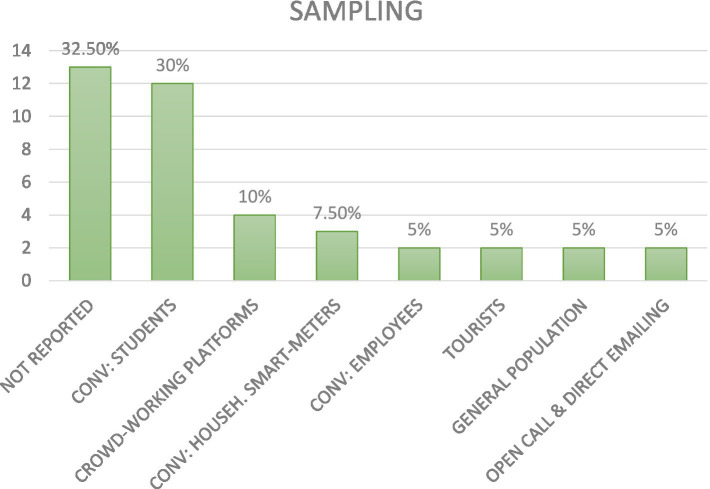
Sampling methods used in the screened articles.

The majority of published articles (64.86%) used an experimental research design (between-subject design: 32.43%, within-subject design: 24.32%, mixed design: 8.10%); a total of 13.51% of studies used a quasi-experimental design (between-subject design: 8.10%, within-subject design: 5.40%); and another cluster emerging from our analysis is one related to field experiments (18.92%). About the sampling method, the following picture emerged (Figure 5).

Most studies^3^ employed convenience sampling techniques used with the following targets: students (30%, voluntarily participation or participation in the exchange of course credits); employees (5%), or clients from households with installed smart-meters (7.50%); crowd-working platforms (e.g., M-Turk) (10%) volunteers from the general population (5%), open call and direct emailing (5%), tourists (5%). Moreover, 32.50% of studies did not report information about the sampling procedure.

### 3.3 RQ 3: technology type

**Figure 6:**
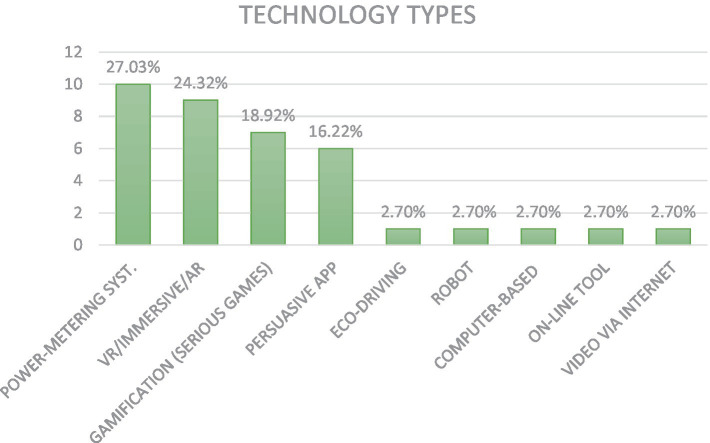
Technology type used to promote SBs.

About the digital technologies used to promote environmental sustainability, the following picture emerged (see Figure 6).

The digital technologies used to promote the SBs investigated were the following: Power-metering system (27.03%); Virtual Reality/Immersive Reality/Augmented Reality (24.32%); Gamification (18.92%) and Persuasive mobile app (16.22%); Eco-driving (2.70%); Video administered via the internet (2.70%); Computer-based learning environments (2.70%); On-line tools (2.70%); and Persuasive robots (2.70%). In the following paragraphs, evidence that emerged for each digital technology is described.

#### 3.3.1 Power metering systems

Power metering systems include IT systems used to measure resource consumption (e.g., electricity, gas, water) and make this information accessible to the user, such as (a) *smart meters* (IT systems collecting the electricity consumption at a high frequency and transmitting it to a data hub); (b) *smart shower-meters* (IT systems providing feedback on energy and water consumption in real-time); and (c) *eco-feedback technologies* (technology that provides feedback on individual or group pro-environmental behaviors to manage environmental impact). Empirical evidence about these three kinds of systems is addressed in the following paragraphs (see Table 1).

**Table 1:** Studies employing smart-meters technologies.

	Authors	Sample	Journal title	Experimental design	Technology type	Outcome measures	Moderators	Results	Intention/Behavior
1	Chiu et al. (2020)	*N* Students = 30 (*N* females = 10, *N* males = 20, aged 21–24)	Journal of Cleaner Production	Within subject (1 condition per week): “No information provided” (no feed-back) vs. “Personal information on electricity compared with peers” (actual individual eco-feedback); “Providing information on sustainable use of electricity” (persuasive eco-feedback); “Providing information on electricity compared to peers and giving awards for sustainable use” quarter (group eco-feedback and reward).	Smart/intelligent devices, eco-feedback and persuasive strategies	1. Behavioral intentions for sustainability 2. Energy consumption	/	+ Successful reduction of personal use of electricity by more than 50% of the participants. + “Providing comparative personal power usage information” had the greatest effect: 67% reduced their usage. = No significant difference between “Providing personal power usage information” and “No information provided.”	Behavior
2	Henn et al. (2019)	*N* Partecipants = 186 (23.7% females; 5.4% did not indicate their gender; *M* age = 56.5, *sd* = 11.0)	Journal of Environmental Psychology	Quasi-experimental between subject design: voluntarily registered to smart-meter feed-back program (*N* = 127) vs. not registered (*N* = 59)	Smart-meter based feed-back interventions	1. Amount of energy saved in kWh (observed period 2008–2012)	(a) Environmental attitude	+ Increased energy savings for the ones registered in the smartemeter program, especially for the ones with higher environmental attitude levels (moderation effect).	Behavior
3	Schwartz et al. (2015)	N Households = 7 (2 Couples, 2 Single, 3 Families), *N* Participants = 16 (*N* females = 9, N males = 7; aged 5–66, *M* age = 33.06)	Interacting with Computers	Longitudinal qualitative study (18 months)	Feedback systems in Sustainable Interaction Design	1. Reduction in electricity consumption	/	+ 7.8% average reduction in electricity during a period of 18 months when compared with the consumption in the year before the HEMS deployment.	Behavior
4	Hafner et al. (2019)	*N* Partecipants = 599 (*N* females = 390, N males = 202, N undisclosed = 2, Missing = 5, aged 18–72, age median = 34)	Environmental Education Research	2 (normative information: yes vs. no) x 2 (feedback type: financial vs. environmental) and a control condition (random assignment) who received no normative information and no feedback information	Feedback via internet	1. Pro-environmental decision-making behavior 2. Pro-environmental purchasing	/	+ There was low uptake of the heat pump in the control condition where no frame information was provided. Yet, in both conditions where normative information was provided, respondents were significantly more likely (vs. control) to choose the heat pump, advancing understanding of normative social influence in the context of pro-environmental purchase behavior. When normative information was not provided, only financial but not environmental, feedback was associated with a significantly greater likelihood of heat pump choice. = The main effect of normative information was replicated for behavioral intentions (though only for homeowners), but there was no norm-feedback interaction (regardless of homeownership).	Behavioral intention
5	Casado-Mansilla et al. (2020)	*N* Partecipants Pre = 303; *N* Partecipants Post = 105	Energies	Experimental within subject design (pre-post)	GreenSoul (GS): smart-meter with personalized feed-back	1. Promotion of pro-environmental behavior 2. Promotion of energy conservation	/	+ The three most effective Persuasive Principles (PPs) for changing energy-related behavior in a working environment are “Cause and Effect,” “Conditioning,” and “Self-monitoring.”	Behavior
6	Tiefenbeck et al. (2018)	*N* Households = 620	Management Science	Framed field experiment	Smart shower meter	1. Promotion of pro-environmental behavior 2. Promotion of energy conservation	/	+ Real-time feedback reduced resource consumption for the target behavior by 22%. High baseline users displayed a larger conservation effect, in line with the notion that realtime feedback helps eliminate “slack” in resource use.	Behavior
7	Tiefenbeck et al. (2019)	*N* Hotels = 6, *N* rooms = 256, *N* observations = 19,596	Nature Energy	Field Experiment in collaboration with 6 hotels (randomized control trial)	Real-time feedback	1. Promoting energy conservation behavior	/	+ The treatment group used 0.215 kWh less energy each shower than the control group, which used 1.883 kWh. This indicates a 11.4% decrease. When flow rate is controlled, the effect is still quite significant, with a decrease of 0.188 kWh, or 10.0%.	Behavior
8	Pereira-Doel et al. (2019)	*N* showers = 1.962 (*N* treatment group = 1.048, *N* control group = 914)	e-Review of Tourism Research	Covered Field Experiment between subject design; treatment (guests could see the timing for their ongoing showers in the Display) vs. control (the Display was not installed and hence the timing could not be seen, so guests were not nudged)	Real-time feedback provided by smart water-saving technology (trough Aguardio)	1. Pro-environmental behaviors	/	+ The Aguardio effect of this intervention is large and highly significant: guests who received real-time feedback used on average 40.91 less seconds (mean = 4 min 58 s) than guests in the control group (mean = 5 min 39 s). This represents an average reduction of 12.06%. The independent t-test showed that the difference between groups was statistically significant.	Behavior
9	Chen and Sintov (2016)	*N* Partecipants = 856 (54.9% females; *M* age = 47.1, *sd* = 17.8)	Energy Research and Social Science	Survey	Website, mobile phone application, in-home display	1. Encouraging connection with nature 2. Promotion of pro-environmental behaviors 3. Promotion of energy conservation 4. Adoption of sustainable innovations	/	+ According to the regression results, nature connectedness was the best predictor of all outcomes, with higher nature connectedness predicting a higher chance of technology and program uptake. The connection with nature can aid in “bridging the logic gap” between the adoption of sustainable innovations and environmental conservation.	Behavioral Intention
10	Mozo-Reyes et al. (2016)	/	Resources, Conservation and Recycling	Experimental design - Mixed methods (three experiments divided by length: 2 days, 1 week, 1 month)	Eco-feedback technology	1. Pro-environmental behaviors 2. Promote recycling activities	/	+ Two day experiment: 95% probability that the number of visits (persons who used the bin torecycle) and recycled items were significantly higher at the WeRe-cycle Bin than the baseline bin. One week experiment: the highest recycling rate in one bin duringthe baseline week was significantly lower than the recycling on theWeRecycle bin. One month experiment: 99% probability of increased recycled items during theWeRecycle intervention.	Behavior

##### 3.3.1.1 Smart-meters

Chiu et al. (2020) conducted a between-subject experiment with smart electronic sockets to collect the electricity use information on a personal computer, and a mobile app to register the measured data instantly. This intervention obtained the following results: (a) a reduction of personal use of electricity by more than half of the participants; (b) greater efficacy for the “Providing comparative personal power usage information” experimental condition: 67% of participants reduced their electricity use (Chiu et al., 2020).

The role of smart-meter-based feedback interventions was investigated also by Henn et al. (2019) in a quasi-experiment conducted in Germany, where a local energy supplier provided smart-meters to all its customers’ households. Participants could access a web-based feedback portal with individual information about their household’s electricity use. This latter was provided in different formats: per day, per quarter year, or as a trend (whether electricity use was higher or lesser compared with previous levels of consumption: 2008–2012). It was found that only people who held a certain level of environmental attitude, like having rigor in using the provided information, saved more energy in the feedback portal compared to the control group (Henn et al., 2019). In other words, a passive attitude- i.e., registering for a web portal - not sufficient but active involvement is needed.

A living lab setting of seven households lasting 18 months was instead developed by Schwartz et al. (2015) for implementing a Home Energy Management System (HEMS) that provided feedback through TV, PC, smartphones, and tablet-based interfaces. The Authors found a 7.8% drop in electricity consumption compared to the year preceding the 18 months of HEMS. Another study on the role of normative feedback (financial vs. environmental) in promoting pro-environmental decision-making behavior, i.e., home heating (heat pump), was conducted in a 2 × 2 (plus control) choice experiment with a sample of the United Kingdom general public. Participants could choose between a typical heating system (a gas boiler) and a relatively more energy-efficient alternative (heat pump). In the normative information condition, respondents chose the more energy-efficient alternative in both financial and environmental feedback; however, when no normative information was provided, only financial but not environmental input worked in the direction of more energy sustainable choices. The main effect of normative information on behavioral intentions was replicated (albeit only for homeowners), but there was no significant interaction between normative condition (present vs. absent) and feedback type, controlling for home ownership status (Hafner et al., 2019).

A final example, regarding smart-meters, is the GreenSoul (GS) project, conducted in tertiary buildings (Casado-Mansilla et al., 2020). GS is based on monitoring devices of shared and personal equipment, such as Lighting, Heating Ventilator, Air Conditioning, and appliances with different feedback, e.g., creating, enhancing, or confirming an energy-saving behavior. The Authors implemented a recommendation-based intervention through ICT with a pre-post experimental design showing that the three most effective Persuasive Principles (PPs) in a working environment were “Cause and Effect,” “Conditioning,” and “Self-monitoring” (Casado-Mansilla et al., 2020).

##### 3.3.1.2 Smart shower meters

In a large-scale field experiment, Tiefenbeck et al. (2019) provided real-time feedback to participants on their showering habits. The intervention lowered water use for the target behavior by 22%, resulting in substantially greater absolute conservation gains compared to traditional policy interventions, based on aggregated feedback. The method is inexpensive, technically suited to the great majority of families, and yielded daily savings of 1.2 kWh for each household, which is greater than the average energy use for lighting. This intervention showed how digitization could make information available to energy users, allowing them to overcome salience bias and behave according to their preferences (Tiefenbeck et al., 2019). In another large-scale field experiment conducted by Tiefenbeck et al. (2019), participants received real-time feedback on their showering activity to encourage water consumption reduction. Results showed that treatment effects were large (11.4% reduction in water use), indicating substantial water conservation among participants who did not opt-in and in a context where participants were not financially responsible for energy costs.

Another study demonstrating the effectiveness of real-time feedback provided by smart water-saving technology for shortening shower time was the field experiment run by Pereira-Doel et al. (2019) who showed a 12.06% reduction in showering time (*N* = 1.962) confirming that real-time feedback is efficient in stimulating sustainable behavior choices. Chen and Sintov (2016) analyzed the intentions to adopt energy management technologies and programs (e.g., a free website, smartphone app, and in-home display monitor) to handle home electricity consumption. Findings revealed that nature connectedness, alongside digital technology use, was associated strongly with all measured outcomes, suggesting that a positive relationship with nature might help bridging the logical gap between the adoption of sustainable innovations and environmental protection.

##### 3.3.1.3 Eco-feedback technologies

Mozo-Reyes et al. (2016) used a low-cost, low-energy electronic recycling bin design named “WeRecycle bin”, that is programmed on human-computer interaction and social fundamentals to provide persuasive eco-feedbacks. Using mixed-methods research, the WeRecycle bin obtained positive responses in three different experiments, with different social settings and exposure time, demonstrating impacts for public recycling. The investigated studies about power-metering systems showed the great potential of these technologies in promoting SBs in the building residential sector, which is by far the world’s largest energy consumer and emitter. Indeed, residential buildings offer great potential and cost-effectiveness in improving energy efficiency and carbon mitigation, consistently with the Paris UN Conference climate goals (Bastida et al., 2019; Chen et al., 2023; Xiang et al., 2023; Zou et al., 2023).

Power-metering could also potentially provide solutions to some of the challenges faced by the energy industry: decarbonization, decentralization, and digitalization, in order to empower European Union consumers (Andoni et al., 2019). Moreover, such technologies can be easily adapted to multiple contexts, both public and private, like schools, hospitals, offices, and universities.

#### 3.3.2 Virtual reality

The term *virtual reality* (VR) refers to “a wide variety of computer-based applications commonly associated with immersive, highly visual, 3D characteristics that allow the participant to look about and navigate within a seemingly real or physical world” (Lopreiato, 2016, p. 40). It has received great attention for its ability to increase the individual’s sense of presence, which is commonly described as the feeling of “being there” (Grassini et al., 2020) which is supposed to influence emotions and, in turn, allow a better connection to the target situation (see Table 2).

**Table 2:** Studies based on virtual reality.

	Authors	Sample	Journal title	Experimental design	Technology type	Outcome Measures	Moderators	Results	Intention/Behavior
11	Oh et al. (2020)	*N* Students = 76 (*N* females = 53, *N* males = 23, aged 18–28, *M* Age = 19.57)	Science Communication	Lab Experiment (between-subjects design): 360-degree condition vs. unidirectional condition	Virtual environment: 360 degree video	1. Perceived Interactivity 2. Self-efficacy to protect the environment 3. Behavioral intention to protect the environment (promotion, prevention) 4. Message Perception (fun, credibility) 5. Issue Involvement	(a) Environmental Self-efficacy	**+** Enhancement of perception of modality interactivity and in turn greater perceived fun, (but not credibility) and intention to protect the environment in terms of promotion (but not prevention).**+** Increased perceived fun and credibility of the video content and greater reduction intention to protect the environment only for high environmental self-efficacy participants (moderation effect).	Behavioral intention
12	Soliman et al. (2017)	*N* Partecipants = 227 (*N* females = 158, *N* males = 67, *N* other = 1, *N* undisclosed = 1, *M* age = 21.20, *sd* = 6.42)	Journal of Media Psychology	Experimental design: 2 (video: natural vs. built environment) x 2 (display device: desktop screen vs. headmounted display)	Immersive technology	1. Nature Connectedness (Inclusion of nature in the self, Connectedness to Nature Scale) 2. Pro-environmental behaviors	/	**+** Influence of type of video but not of technology on Nature Connectedness;**=** no influence on three different observed pro-environmental behaviors.	Behavior
13	Nelson et al., 2020	*N* Participants = 1,006 divided in: *N* students = 487 (55% females, *M* age = 20.4, *sd* = 3.55); *N* general public = 248 (47% females, *M* age 23.2, *sd* = 9.22); *N* tourists = 271 (55% females; *M* age 29.3, *sd* = 10.25)	Plos One	2 (framing: positive vs. negative) x 2 (experimental design: 360-degree condition vs. unidirectional condition) and control condition	Virtual Reality	1. Donations to a conservation charity organization, Emotions, Sense of presence	/	**+** Both positive and negative framing, and low and high visual immersion significantly affect donations to a charity in comparison to no media communication.**=** No significant differences between experimental condition in the intention to donate to a charity organization;**+** Both positive and negative framing, and low and high visual immersion significantly affect emotional responses and sense of presence in comparison to no media communication (with the highest scores in the 360° VR treatment).**+** Among the tourist sample the 360° film with a negative message evoked the larger amount of donations.	Behavioral intention
14	Ahn et al. (2014)	1. *N* Students = 47 (*N* females = 29, N males = 18, aged 18–46, *M* age = 21.60, *sd* = 4.27); 2. *N* = 60 (*N* females = 45, *N* males = 15, aged 18–24, *M* age = 20.32, *sd* = 1.16)	Computers in Human Behavior	1. Experimental design (between/within subject): IVE (*N* = 24) vs. print (*N* = 23) version; 2. Experimental design (between subject): IVE (*N* = 21) vs. print (*N* = 20) vs. video version (*N* = 19) in two times (T1 and T2 after one week)	Immersive Virtual Environment	1. Environmental locus of control, observed Environmental behavior (i.e., number of napkins used to clean a table) 2. Environmental locus of control, Environmental behavioral Intention, measured Environmental behavior	/	Study 1.**+** Participants who embodied the experience of cutting down a virtual tree used 20% less napkins compared to participants in the print condition.**+** There was a significant increase in internal environmental locus of control immediately following both experimental treatments. Study 2.**+** The effect of IVEs on environmental locus of control and behavior was consistently stronger than print and video at T2.**=** The effects between print and video were not significantly different across all measures at T2. The relationship between experimental conditions and environmental behaviors was mediated by environmental locus of control, but only at T2.	Behavior
15	Chirico et al. (2021)	*N* Students = 60 (*N* females = 35, *M* age = 26.63, *sd* = 11.56; N males = 25, *M* age = 31.24, *sd* = 15.94)	Virtual Reality	Experimental between-subject design: 1. numerical: presenting statistical evidence of plastic consumption in numeric form; 2. concrete: presenting statistical evidence of plastic consumption through heaps of 3D plastic objects; and 3. mixed: numerical and concrete	Virtual reality	1. Emotions, Sense of presence, Attitute toward the environment, Attitude toward plastic (waste and consumption, use and recycle), Enviromentally Responsible Behaviors	/	**+** Participants in the concrete and in the mixed condition reported higher levels of all the variables investigated compared to the numerical format.**-** Numerical format was the least effective across all dimensions.**=** Concrete and mixed formats were similar.	Behavioral Intention
16	Bailey et al., 2015	*N* Students = 70 (47.1% females, 52.9% males, aged 18–22, *M* age = 18.4)	Environment and Behavior	Between Subject Experimental Design: 2 (vivid vs. not vivid scenario) x (personal vs. not personal scenario)	Immersive virtual reality	1. Water temperature 2. Water amount	/	**+** Cooler water use in the vivid condition;**=** No significant effect of personal messages on water temperature;**=** No interaction effect;**=** No significant effect on water usage.	Behavior
17	Fox et al. (2020)	*N* Students = 190 (*N* females = 112, N males = 78, aged 18 to 34, *M* age = 20.25, *sd* = 2.41)	Telematics and Informatics	Experimental (longitudinal between subject design): 2 (psychological distance: near vs. far) x (risk perception: contingent vs. not contingent)	Virtual environments	1. Time 1 Environmental Behavior; 2. Time 2 Policy Support	/	**-** Experiencing a close (*vs* distant) environment lead to lower psychological distance and in turn **+** Higher perceptions of risk, which lead to**+** more environmental behavior and **+** policy support.**+** Experiencing a contingent (*vs* non-contingent) environment lead to higher perception of**+** interactivity and in turn**+** higher self-efficacy, which lead to**+** more environmental behavior and **+** policy support.	Behavior
18	Coghlan (2020)	*N* = 75	Journal of Sustainable Tourism	Action design research	Virtual reality interpretive game	1. Pro-environmental reward after the game (e.g., $10 donation to the Cairns Turtle Rehab Centre) vs. two no pro-environmental selection	/	**+** More donations (72% for the game and 60% for the control VR video).	Behavior
19	Coen et al., 2019	*N* students = 8	Human Affairs	Experimental design (within subject)	Augmented reality	1. Pro-environmental; 2. Environmental awareness	/	**+** Improvements of SBs in 7 domains (cost of having single/double glazed windows, electricity consumption, washing machine water temperature, CO_2_ emissions, LED/traditional incandescent light bulbs, shower water conservation, insulation vs. no insulation).**+** Increase in environmental awareness.	Behavior
20	Ahn et al. (2015)	*N* = 114 (N females = 80, *N* males = 34, aged 18–32, *M* age = 20.80, *sd* = 2.05)	Communication Research	Mixed factorial design (T1, T2, T3) and between subject design: control (*n* = 21), loss x high interactivity (*n* = 26), loss x low interactivity (*n* = 21), gain x high interactivity (*n* = 25), or gain x low interactivity (*n* = 21)	Virtual environments	1. Environmental response efficacy 2. Environmental behavioral intention 3. Environmental behavior (napkins used to clean a table)	/	**+** More napkins were used by participants in the control condition compared to those in experimental ones. Thus, framed virtual experiences succeeded in promoting environmental behavior immediately after exposure. **+** Gain framed experiences elicited greater environmental behavioral intentions than loss framed experiences immediately after the exposure and also after 1 week (Time 2) **=** No significant difference at time 3 (Time 3)	Behavior

In VR research related to SBs, we can distinguish studies using different tools, such as 360-degree videos, immersive virtual environments (IVEs), video games with vividly rendered settings, and augmented reality (AR). Each tool is addressed in the following paragraphs.

As regards the 360-degree video feature, it offers a full panoramic view of the scenery, as the user manages the video’s perspective (Feng, 2018). 360° videos became one of the most promising tools to communicate news and scientific information due to their capability to offer a highly realistic media experience and accessibility (Schild, 2020). This technology was used in video presentations in a controlled laboratory setting to stimulate the participants’ environment protection and content perception (Oh et al., 2020). Results showed an enhancement of the perception of modality interactivity and, in turn, greater perceived fun (but not credibility), as well as intention to protect the environment in terms of promotion (but not prevention) in the 360° video compared to unidirectional videos (Oh et al., 2020). A similar experiment was realized in a laboratory setting where the exposure to a video on natural vs. built environments was offered with a head-mounted display compared with a traditional computer monitor (Soliman et al., 2017). Results showed that the content of the video mattered and influenced attitude toward nature (built vs. natural environment), while the type of technology (computer monitor vs. head-mounted display) had no impact. Moreover, experimental manipulation increased the perception of nature connectedness but it did not meaningfully impact different pro-environmental behaviors. Lastly, technology type (desktop or less immersive or head-mounted display or more immersive) did not influence the dependent variables. VR was also used in a 2 (positive vs. negative framing) x 2 (unidirectional video vs. 360° video) field experiment about the coral reef crisis showing that visual communication increases the amount of donation to a charity organization (Nelson et al., 2020). The Authors suggest that risk communication can be tailored according to different targets. In their studies, they recruited different samples (students, the general population, and tourists): in particular, among the tourist sample the 360° film with a negative message evoked a larger amount of donations. In conclusion, all treatments significantly influenced emotions and the sense of presence in comparison to no media communication (with the highest levels in the 360° VR condition).

Regarding Immersive Virtual Environments, these are digital devices based on vivid layers of sensory information in a simulated environment (Blascovich and Bailenson, 2011). Ahn et al. (2014) conducted two different studies to analyze the power of embodied experiences within IVEs by comparing the effects of cutting a virtual tree vs. reading a print description of the same operation (Study 1) or watching a video about tree-cutting to promote paper conservation (Study 2). In IVEs users may feel negative future consequences of actions, like the personal contribution to deforestation, as if they were occurring in the moment of simulated experience. The simulated experience was very vivid: spatialized audio information was conveyed through earphones to mimic realistic sounds of the forest; moreover, the computer was also equipped with a force-feedback haptic joystick (Sensable Phantom Omni) to allow real-time interaction with objects in the IVE. In Study 1, there was a significant increase in the internal environmental locus of control in both experimental conditions. The observed behavioral measure chosen was napkins usage: participants in the IVE condition used 20% fewer napkins immediately after the treatment in comparison to the ones in the print condition. In Study 2, interesting effects were observed after 1 week (at T2): the effect of IVEs on environmental locus of control and behavior was consistently stronger compared to the other conditions. In another study conducted with VR, Ahn et al. (2015) investigating the impact of receiving any kind of environmental message about deforestation via IVEs, showed a reduction of actual paper consumption by approximately 25% of participants. Furthermore, presenting a gain scenario, i.e., a vivid virtual experience of growing a tree vs. a loss scenario, i.e., the experience of cutting a tree, increased environmental response efficacy immediately after the treatment, which in turn led to greater intentions to engage in pro-environmental actions. One week after the experimental exposure (T2), the effect remained stable; however, framing did not affect self-reported pro-environmental behavior in the longer run, so after 2 weeks (T3). In another study, Chirico et al. (2021), conducted a laboratory experiment to understand how different statistical information formats, i.e., numerical, concrete, and mixed, can enhance VR’s persuasive potential for plastic consumption, recycling, and waste. In the IVEs statistical evidence was represented using a visual representation of the corresponding amount (i.e., displaying 10 bottles instead of the number 10) to render the information more concrete. Participants in the concrete and mixed conditions reported higher levels of sustainable behavioral intention compared to the numerical format, while concrete and mixed formats were similar. Another experiment was realized to investigate the impact of vivid and/or personal messages on hot water savings behavior (Bailey et al., 2015). Participants experienced a virtual shower while wearing a fully immersive virtual reality helmet, in which they received feedback on the amount of energy used to heat and transport the virtual water (with various levels of vividness and/or personalization). The feedback was represented as coal to make energy use consumption more concrete and to represent the harmful effects of energy use due to its association with climate change. Results showed that participants exposed to vivid messages (i.e., images of coal) used a cooler water temperature compared to those exposed to the textual ones.

Video games with vividly rendered settings are also considered Virtual Environments (VEs). In this regard, Fox et al. (2020) tested the effects of a serious game that allowed users to engage in a simulated environmental clean-up. The study examined how manipulations of psychological distance (near vs. far) and interactivity (contingent vs. non-contingent) could influence SBs. In the VE participants navigated down with an avatar in a kayak a polluted river that was described as geographically and temporally close or distant. The river was surrounded by a forest-like 3D environment in which sounds of nature such as flowing water and chanting birds were audible. Experimental manipulations of psychological distance and interactivity influenced perceptions of environmental risk, self-efficacy, policy endorsement, and behavioral intention during the serious game. In the near condition participants perceived a higher perception of environmental risk, which led to greater policy endorsement as well as more SBs outside of the lab setting and at a different time point. Regarding interactivity, in the contingent condition, in which the players experienced immediate consequences of their actions, there were higher levels of environmental efficacy compared to the non-contingent version. Effects remained consistent outside of the lab setting and at different time points, like the ones registered for psychological distance. A case study conducted by Coghlan (2020) explored a ‘‘build your own reef’’ game with tourists, using VR to simulate an immersive, 3D underwater seascape. In this simulated environment, tourists could emulate a reef they had previously seen, building it up while recognizing the marine elements, like corals and fishes that they had already seen, and trying to know them while answering questions about the marine life that they experienced. Results showed that there was a slightly higher pro-environmental choice in the experimental condition: playing the game brought more donations in comparison to watching a video on the same topic (72% for VR based serious the game and 60% for the control VR video). Concerning AR, it is the real-time merging of virtual three-dimensional objects with the actual environment, representing an advancement of (VEs). Unlike traditional VEs, AR allows users to interact with virtual elements in a completely artificial manner, taking place within the real environment (Coen et al., 2019). What sets AR apart is that virtual objects are overlaid onto real ones, seamlessly integrating them into the context of the physical world. By superimposing digital information onto existing information, the realness of the physical world is expanded, creating an enhanced perception for the user. AR applications utilize a technique called Simultaneous Localization and Mapping (SLAM) to determine the position and orientation of the rendering device relative to the surroundings. To determine the position and orientation of an object within its environment, a combination of sensor-based techniques (such as accelerometers and gyroscopes) and vision-based techniques (involving cameras, depth sensors, and artificial intelligence for recognizing visual markers or mapping and learning objects and the surrounding area) are often employed (Coen et al., 2019). Furthermore, certain AR systems take advantage of Bluetooth markers, Wi-Fi signal triangulation, and GPS data to accurately locate the rendering device within a given context. In this regard, Coen et al. (2019) found that the use of mobile technology (Augmented Reality) has positive consequences for both individual users in terms of improvement of SBs in seven different domains (cost of having single/double glazed windows, electricity consumption, washing machine water temperature, CO_2_ emissions, LED/traditional incandescent light bulbs, shower water conservation, insulation vs. no insulation) and increase of environmental awareness.

#### 3.3.3 Gamification

Gamification is an emerging field that aims to capture attention and engage people in sustainability topics. While the definition of gamification is still evolving (Seaborn and Fels, 2015), it generally refers to the integration of game design elements, such as points, leaderboards, levels, narrative, or time constraints, into non-game contexts, beyond mere entertainment purposes. The power of gamification lies in its ability to motivate players and create emotional connections within a gamified setting, whether the topics are for pleasure, education, or of societal importance. Gamification encompasses serious games and geo-games Dicheva et al. 2015. Serious games are designed with a purpose that extends beyond pure entertainment and aims to influence thoughts and actions in real-life situations (see Table 3).

**Table 3:** Studies based on Gamification technologies.

	Authors	Sample	Journal title	Experimental design	Technology type	Outcome measures	Moderators	Results	Intention/Behavior
21	Martínez-Borreguero et al. (2020)	*N* Students = 81 (aged 14–16)	Sustainability	Quasi-experimental type: within subject design (pre-test, post-test) and between subject design: Didactic interventions: 1. WebQuest (*N* = 28), 2. Game about climate change (*n* = 27), 3. Game about electricity (*N* = 26)	Didactic ICT interventions: Web-Quest and Serious Games	1. Knowledge 2. Environmental Behavior 3. Environmental Attitude	/	**+** The block with the greatest group progress was that of sustainability, followed by the ones on renewables and saving measures.**+** All the groups obtained an improvement from pre-test to post-test in terms of the level of knowledge in the sustainability block. Likewise, an improvement was also observed, although to a lesser extent than in the previous case, in relation to the block on renewable energies (group B obtained the better score). Finally, concerning the block of savings measures, it can once again be seen that it was group B that improved its level of knowledge the most.**+** The use of ICT-based active methodologies promotes environmental behavior in students.**=** The intervention has produced any change in the environmental attitude of the students.	Behavioral Intention
22	Moore and Yang (2020)	1. *N* Partecipants = 61 (*N* females = 36, *N* males = 25, *M* Age = 20.28, *sd* = 1.72); 2. *N* on-line participants = 293 (*N* females = 128, *N* males = 165, *M* Age = 38.83, *sd* = 11.19)	Environmental Communication	Experimental (within/between subject design): 1. Serious game vs. Trailer of the game vs. control; 2. Trailer vs. Control	Serious game/single- or multi-player	1. Eco-guilt, pro-environmental choice, intention toward future environmental behavior, two different observed behaviors (e.g., water bottle choice) 2. Eco-guilt, pro-environmental choice, intention toward future environmental behavior	(a) Environmental Attitude, (b) Trait Empathy in both studies	Study 1:**+** increase of one observed pro-environmental behavior (i.e., refusal of not re-usable water bottle and so not sustainable) in the trailer condition; Study 2: greater intention to engage in future environmental behaviors only for the ones in the trailer condition with lower levels of Environmental Attitude (moderation effect).	Behavior & Behavioral Intention
23	Reihana et al. (2019)	*N* = 8, aged 12–16	New Zealand Journal of Ecology	Within subject design (pre-post)	Serious Game: Eko.	1. Eco-literacy 2. Indigenous knowledge	/	**+** 65% of the students reported some degree of knowledge acquisition, with 28% citing no recall, and 35% recalling at least 3 species from the potential 15 species which they were exposed to.	Behavioral Intention
24	Casals et al. (2020)	*N* social houses with monitoring system deployed = 88	Energy and Buildings	Between Subject designs: Experimental (*n* = 44) and Control (*n* = 44); Within subjects design: pre-post	Serious game and metering system	1. Energy consumption 2. Energy consumption behavior and energy awareness 3. Peak demand 4. Social media activity and energy knowledge sharing 5. IT literacy	/	**+** The intervention increased social housing tenants’ awareness and engagement in certain energy saving behavior and provided an average electricity saving of 3.46% and an average gas saving of 7.48%. Although savings were found not to be sta-tistically significant, an effect size was detected (0.2).	Behavior
25	Schaal et al., 2018	*N* students = 206 (52.9% females; *M* age = 13.7, *sd* = 2.3)	International Journal of Science Education, Part B	Quasi experimental design; within subject (pre-post)	Geogame for experiential outdoor learning	1. Attitudes toward nature 2. Biodiversity-related knowledge	/	**+** Game-related enjoyment increased positive attitudes toward nature.**-** game-related enjoyment did not increase biodiversity-related knowledge	Behavioral Intention

Sustainability is one area where serious games are widely applied, with goals including raising awareness of sustainability challenges, imparting knowledge and understanding, and encouraging players to develop environmentally and socio-economically harmonized solutions (Ouariachi et al., 2019). For instance, two different serious games were developed in an experimental lab study for designing and validating ICT-based learning interventions for sustainability content (Martínez-Borreguero et al., 2020). During the study, participants were exposed to three different experimental conditions: a Webquest and two distinct serious games. In one experimental group, the serious game “My Green Energy Planet” was utilized, an online video game focused on climate change. In this game, participants were tasked with managing a city by implementing energy-saving measures, utilizing renewable energy production plants, and avoiding non-renewable energy plants.

Another serious game called “Control of the Spanish Network,” developed by the institution Red Eléctrica de España, was employed in the study. The results indicated that all groups showed improvement in knowledge related to sustainability from the pre-test to the post-test. The overall utilization of active ICT-based methodologies effectively promoted environmental behavior among the students. However, the intervention did not result in any change in the environmental attitudes of the students. In a separate study, Moore and Yang (2020) utilized a serious game named Eco, which could be played both online and offline, either as a single-player or multiplayer game (Strange Loop Games, 2015). Eco provides a collaborative space where players work together to save the world from an impending meteor while simultaneously maintaining the ecosystem. What sets Eco apart from other open-world games is its unique feature of resource depletion. Unlike games that require players to hunt endlessly for survival, Eco introduces the concept of resource balance, where overhunting and other activities can potentially lead to the extinction of resources, such as driving the deer population to extinction. The effectiveness of Eco was evaluated in two separate studies. In the first study, Eco was compared to a video of the game or a trailer, as well as a control condition. Only the trailer condition showed an increase in observed pro-environmental behavior, such as using reusable water bottles. In the second study, no significant differences were found between the video condition and the control condition. Moore and Yang (2020) also examined the moderating role of environmental attitude in the relationship between experimental conditions and behavioral intention. It was discovered that individuals with lower levels of positive environmental attitude exhibited a greater intention to engage in future environmental behaviors when exposed to the video condition or the game trailer.

A further similar serious game called Eko was employed in a field experiment conducted in New Zealand (Reihana et al., 2019). By incorporating fundamental ecological concepts with indigenous Maori knowledge, the game successfully fostered eco-literacy, indigenous knowledge, and pro-environmental behaviors. Among the participants, 65% reported some level of knowledge acquisition, with 28% indicating no recall, and 35% recalling at least 3 out of the 15 species they were exposed to. Another serious game was developed to encourage energy efficiency and reduce carbon emissions among social housing tenants (Casals et al., 2020). The game underwent validation in a sample of European social housing units using a two-stage experimental design that incorporated pre-post and control group approaches. The game’s protagonist is the Energy Cat, controlled by the player, who resides in a house with human characters controlled by artificial intelligence. The Energy Cat strives to live in a comfortable and energy-efficient house, guiding the human characters to modify their energy-consuming behavior. The serious game proved effective in enhancing social tenants’ understanding and involvement in energy efficiency, resulting in an average electricity saving of 3.46% and an average gas saving of 7.48%.

Regarding geo-games, they encompass location-based and location-dependent games (Schaal et al., 2018) that require players to engage in activities and movement in real-world settings. These games often incorporate narratives and game characters to create an immersive experience. Smartphones, commonly used for geo-games, provide unique capabilities for game tasks and location-based learning. The integrated camera, microphone, texting, and video features of smartphones can be utilized to provide necessary information within the game or to document observations and answers. Additionally, GPS and integrated map services enable the planning of guided tours (Horton et al., 2013). As a result, geo-games offer extensive opportunities for environmental education and the promotion of sustainable behaviors. Schaal et al. (2018) developed a geo-game that aimed to create sensory experiences and foster appreciation for biodiversity through mobile technology. The results indicated that game-related enjoyment led to more positive attitudes toward nature, although it did not significantly increase biodiversity-related knowledge.

#### 3.3.4 Persuasive apps

As computing technology gets more powerful and transportable, it becomes more and more integrated into people’s everyday lives and influences their behavior. Technology may be created specifically to influence behavior and, in this vein, it has been referred to as “persuasive technology” (Filippou et al., 2015; see Table 4).

**Table 4:** Studies based on persuasive apps.

	Authors	Sample	Journal title	Experimental design	Technology type	Outcome measures	Moderators	Results	Intention/Behavior
26	Mahmud et al. (2020)	*N* Students = 48	Sustainability	Quantitative/Qualitative study, Quasi-experimental design (*N* treatment group = 28, *N* control group = 20)	Online gamification activities	1. Sustainability knowledge 2. Pro-environmental behavior 3. Recognition by the teacher and peers 4. Competition 5. Sense of belonging to a group 6. Time constraint 7. Boredom	/	**+** The treatment group showed, at post-test, a statistically significant difference from the control group in the following factors: sustainability knowledge and pro-environmental behavior.**+** In JouleBug mean points, the treatment group showed a statistically significant difference from the control group.**+** The following factors were identified by the focus group: Recognition by the teacher and peers, Competition and Sense of belonging to a group.**-** Similarly, the focus group showed: Time constraint and Boredom.	Behavior
27	Kimura and Nakajima (2011)	*N* Families = 6, *N* Partecipants = 20 (*N* females = 8, *N* males = 12, aged 15–58)	PsychNology Journal	Longitudinal study (4 weeks) 1. Measurement of ordinary energy consumption before installation of EcoIsland 2. EcoIsland installation and it was requested that only one member of the household use it 3. All family members used EcoIsland 4. All family members used EcoIsland that contains emission- trading system between families.	Persuasive application	1. Environmental ecology awareness 2. Communication within family 3. Cooperation with other participants 4. Competition between participants	/	**+** In the survey, 17 out of 20 participants said that they were more conscious of environmental ecology after the experiment than before.**-** From the air heater electricity usage data, there was no significant correlation with the reported activities.	Behavioral Intention
28	Wemyss et al. (2019)	*N* households = 82	Energy Research & Social Science	Experimental design 2 (location: city 1 vs. city 2) x 3 (gamified structure: competitive vs. collaborative vs. control)	Gamified mobile application	1. Improve electricity consumption behavior 2. Social norms	/	**+** A considerable improvement in electricity savings was reported following the three-month intervention**-** However, one year later, a spillover effect developed, and the beneficial effects were minimal. There was no difference in short- and long-term results between the two separate gamified approaches, competitive group and collaborative group.	Behavior
29	Oppong-Tawiah et al. (2020)	*N* Partecipants = 137	Journal of Business Research	Within-subjects (repeated measures) experiment. The Authors iteratively developed a system, evaluating it in each design cycle (5) including a longitudinal evaluation of PEB (pro-environmental behaviors)	Power-metering, Energy metering sub-system (computer), eco-feedback through virtualized ecological system (garden): mobile application	1. Reduction in electricity consumption 2. Motivation in implementing pro-environmental behaviors	/	**+** Results showed a reduction in electricity consumption and an increase in motivation to continue engaging in pro-environmental behaviors.	Behavior
30	Won (2018)	1. *N* participants = 57 (*N* females = 20, *N* males = 37); 2. *N* Partecipants = 400	Archives of Design Research	Experimental mixed (within-between) Design. 3 treatment conditions (1. Emotional support = Attitude toward Behavior (AB) vs. Subjective Norm (SN) vs. Perceived Behavioral Control (PBC) vs. Behavioral Intention 2. Ability support = AB vs. SN vs. PBC vs. Behavioral Intention 3. Social learning support = AB vs. SN vs. PBC vs. Behavioral Intention) and 1 control (Baseline = AB vs. SN vs. PBC vs. Behavioral Intention)	Applying PSPB (Persuasive Service design strategies based on TPB - Theory of Planned Behavior) design concepts in the development of mobile applications	1. Attitude toward Behavior 2. Subjective Norm 3. Perceived Behavioral Control 4. Behavioral Intention	/	**+** According to social learning support and ability support goals, all three techniques reduced the users’ negative emotions in comparison to the control group and enhanced perceived behavior control and subjective norm (SN).	Behavioral Intention
31	Oliveira et al. (2016)	*N* Partecipants = 12 (*N* females = 4, *N* males = 8, aged 18–22)	Computers in Human Behavior	Within-subjects design AB-BA (Step 1: group A control group and group B intervention group; Step 2: group B control group and group A intervention group)	Persuasive smartphone application	1. Promotion of sustainable behaviors 2. Promotion of energy conservation	/	**+** Participants in all experimental conditions had an increased likelihood to follow the procedures necessary to consume less energy compared to a control condition.	Behavioral Intention

Mahmud et al. (2020) provides an example of the usage of persuasive applications. An online gamified mobile application called JouleBug was utilized in this study to motivate students to improve their sustainability practices through pro-environmental behaviors. JouleBug is a smartphone application with an environmental theme that blends mobile games, social networking, and educational tools to establish and sustain daily habits in environmental education (Mahmud et al., 2020). The Authors conducted a study using a quasi-experimental design and both qualitative and quantitative methods on 48 students, 28 of whom were in the treatment group and 20 were in the control group. The results showed that the treatment group performed significantly better than the control group in the following areas: sustainability knowledge and pro-environmental behavior. Another persuasive gamified application, named EcoIsland, was developed to encourage users to reduce CO_2_ emissions (Kimura and Nakajima, 2011). EcoIsland was designed to be installed in the living room or another prominent place in a household. Kimura and Nakajima (2011) found that the majority of participants stated they were more environmentally concerned after the experiment than before. In contrast, there was no significant correlation with reported activities in the data on air heater electricity use.

Another gamified mobile application was connected to a household’s smart meter to foster a behavioral change in terms of household electricity consumption reduction (Wemyss et al., 2019). In particular, the intervention was designed to activate social norms and improve electricity consumption behavior in neighborhood households. After a three-month intervention, a significant improvement was seen in electricity savings but 1 year later, the survey showed a relapse effect, and the beneficial effects were minimal. There was no difference in short- and long-term results between the two separate gamified approaches, the competitive group, and the collaborative group (Wemyss et al., 2019). In the work environment field, Oppong-Tawiah et al. (2020) created a gamified mobile application to promote sustainable energy use in the office. They used five design cycles to develop and test a system that tracks employees’ electricity usage on their computer-related equipment, engages them through the app using the metaphor of a garden (employees saw in the app a garden that flourished when they conserved electricity), and encourages them to do so. The outcomes of the design cycles reinforced one another, showing how the system lowers employees’ power use and boosts their desire to keep up their pro-environmental behavior.

A further example of a persuasive app is proposed by Won (2018) and was focused on the role of socio-emotional learning and ability support. The results of this research, in which 400 participants were divided equally into four groups including one control group, showed that all three strategies reduced the users’ negative emotions in comparison to the control group and enhanced perceived behavior control, and subjective norm (Won, 2018). Another study conducted with the use of persuasive apps to modify how time appears, i.e., a lowering of time perception, was aimed at reducing excessive energy use (Oliveira et al., 2016). In comparison to a control group, participants claimed that the app increased their likelihood of following the procedures necessary to consume less energy (*ibidem*).

#### 3.3.5 Other categories of digital technologies

Those digital technologies less used in the selected studies are reported in the following lines (See Table 5).

**Table 5:** Studies based on other categories of digital technologies.

	Authors	Sample	Journal title	Experimental design	Technology type	Outcome measures	Moderators	Results	Intention/Behavior
32	Ruscio et al. (2018)	*N* male drivers = 24 (*M* age = 22.2, *sd* = 3.2)	International Journal of Industrial Economics	Between groups design: eco-driving training (n = 12) vs. control (n = 12)	Eco-driving assisting device or EDA	1. Driver’s Behavior 2. Driver’s Acceptance 3. Driver’s workload	/	**+**The experimental group and the control group presented different values of speed profile, with the experimental group keeping a slower speed.**=** No significant differences emerged in the number of gear changes, in the mean time spent for each of the gears, not for the fuel efficiency and the related CO_2_ emissions. = No significant differences emerged in driver’s acceptance and attitudes toward eco-driving.	Behavior
33	Harmon and Gauvain (2019)	1. *N* students = 143 (65% females; aged 18–21; *M* age = 19.44, *sd* = 0.95). 2. *N* participants = 591 (aged 18.76; median age = 25–34); 3. *N* students = 152 (75% females; *M* age = 19.13, *sd* = 1.69)	Basic and Applied Social Psychology	1. Experimental between subject design: a. pro-environmental video (n = 72) b. neutral video (n = 71). 2, Experimental design between subject design: a. pro-environmental video b. neutral video c. reducing disgust video 3, Experimental design between subject design: a. pro-environmental video (n = 72) b. neutral video (n = 71) c. reducing disgust video.	Videos administered via Inernet	(1) a. Water use behaviors, b. Water Attitudes, c. Misinformation, d. Ignorance, e. Social Norms, f. Disgust Sensitivity and g. Environmental Concern; (2) a. Disgust Sensitivity, b. Pro-environmental behavior, c. Willingness to use recicled water. (3) a. Behavioral Intention, b. Behavioral Measures: drinking a bottle of recyvled water and signin a petition about the use of recicled water.	/	Study 1. - Viewing a fact-based video about water as a limited resource dindn’t increase participants’ willingness to endorse sustainable water use.**=** Rates were similar in the treatment and control groups in the Water Attitudes Survey. Study 2. + Viewing Internet video messages about either disgust reactions or water scarcity has a small but unsubstantial effect on people’s willingness to use recycled water. Study 3. + Participants in both experimental conditions reported greater behavioral intention to use recycled water.**=** Viewing pro-conservation videos did not affect actual pro-conservation behaviors. Participants accepted bottled recycled water and signed the recycled-water petition at similar rates.	Behavioral Intention
34	Eggert et al. (2017)	*N* students = 158 students (*N* females = 107, N males = 47 and 4 students who did not specify their gender; *M* age = 17.16)	Research in Science Education	Experimental between subject design: Themes about climate change: 1. Map-generation condition (n = 38), 2. Concepts-provided condition (n = 37), 3. Lines provided condition (n = 41), 4. Concept and lines provided condition (n = 42). Within subject design: pre-post.	Computer-based learning environment	1. Knowledge about the issue of climate change 2. Argumentation processes about possible solution strategies to tackle climate change. 3. Reasoning and decision making on socioscientific issues in general.	/	**=** Increase in students’ competence in the posttest for all four treatment conditions. Thus, all students benefitted from the teaching intervention in a similar way in terms of knowledge gains.**=** Regarding socio-scientific reasoning, students in all four treatment conditions improved on the posttest scores. No differential effects for the different treatment conditions could be found.**=** Students in all treatment conditions improved in a similar way with respect to learning outcomes for “developing solutions.”	Behavioral Intention
35	Ham and Midden (2014)	1. *N* students = 33 (*N* females = 6, *N* males = 27, aged 18–40); 2. *N* students = 120 (*N* females = 48, *N* males = 72, aged 18–40)	International Journal of Social Robotics	1. Experimental between subject design: a. factual feedback condition, b. high-agency social feedback condition, and c. low-agency social feedback condition. 2. 2 (task similarity: low vs. high) × 2 (feedback type: negative vs. positive) mixed model experimental design.	Human–robot interaction	1. Electricity Consumption Score (for both studies)	/	Study 1.**+** Both social feedback conditions had the strongest influence on electricity consumption reduction behavior, compared to the control condition, i.e., a factual feedback about electricity consumption. The strongest effects were obtained especially with negative (*vs* positive) verbal reactions by a domestic robot. Study 2.**+** Task similarity increased the persuasive effects of negative feedback.	Behavior
36	Spence et al. (2014)	1. *N* Participants = 153 (*N* females = 69, *N* males = 84; aged 17–36, median age = 20). 2. *N* Students = 102 (86 females, 16 males; aged 18–21, median = 18)	Journal of Environmental Psychology	1. Between Subject Experimental Design: a. Energy (n = 64), b. Cost (n = 50) and c. CO_2_ (n = 56) Home Energy Calculator conditions. 2. Between Subject Experimental Design: a. Energy (n = 35), b. Cost (n = 32) and c. CO_2_ (n = 35) Home Energy Calculator conditions.	Online Home Energy Calculator	1. a) Social Values, b) Environmental Behavior (donations to a Charity for climate change). 2. a) Interest in reducing energy use, b) Psychological distance of climate change, c) Salience of climate change, d) Salience of financial issues, e) Environmental Behavior (donations to a Charity for climate change), f) Behavioral intention	/	Study 1.**=** The HEC calculator condition did not significantly affect measures of Social Values between time points.**+** CO_2_ and cost conditions differed from one another with the first one being significantly higher, but **=** comparisons of CO_2_ and energy conditions and energy and cost conditions were non-significant. Study 2.**+** Climate change salience was significantly higher in the CO_2_ HEC condition, compared with energy and cost conditions, while the energy and cost conditions did not differ significantly.**=** Amounts of charity donations and behavioral intention did not differ significantly across conditions.	Behavioral Intention
37	Wheaton et al. (2016)	*N* visitors completed pre-tour and post-tour surveys = 362, *N* visitors completing a final survey three months after the tour = 94	Journal of Sustainable Tourism	Quasi-experimental, longitudinal	Technology-based behavioral messaging	1. Promotion of sustainable behaviors 2. Connectedness to nature 3. Environmental attitudes and self-efficacy 4. Commitment to take action	/	The results showed that **=** although the intervention had little influence on conservation action overall,**+** it was effective for a social media-related action.**+** Post-tour conservation actions were significantly affected by emotional connection to wildlife during the tour and repeat visitation to either the same or another state park. + Visitors’ connectedness to nature increased during the three-hour tour, but - returned to pre-visit levels three months later.	Behavior

##### 3.3.5.1 Eco-driving assistance devices

Eco-driving assistance devices aim to reduce CO_2_ emissions by promoting an eco-driving style, which is estimated to result in a fuel consumption reduction of 10 to 30% (Sivak and Schoettle, 2012). To assist drivers in adopting and maintaining eco-driving practices, eco-driving assistance systems (EDAS/EDSS) have been introduced in new vehicles. These systems provide support interventions and feedback to drivers (Ruscio et al., 2018). The effectiveness of eco-driving devices relies on the assumption that the introduction of assisting technology will encourage changes in driving behavior, including smooth accelerations, steady speed, early gear changes, efficient deceleration, and moderate brake usage. In a study conducted by Ruscio et al. (2018) using a virtual driving simulator, the first-time use of eco-driving assistance technology was evaluated in terms of its impact on CO_2_ reduction. The study recorded actual driving parameters and CO_2_ emissions, comparing them to the optimal eco-driving style. Results showed that the initial exposure to eco-driving technology led to a reduction in cumulative fuel consumption, primarily attributed to speed reduction. However, the overall CO_2_ emissions of the experimental group did not differ significantly from the control group.

##### 3.3.5.2 Videos administered on-line

Harmon and Gauvain (2019) explored the effects of online videos on water conservation behaviors. Participants viewed two brief videos–one focused on the global scarcity of water and the other highlighting disgusted reactions to certain conservation methods. While the study found a small positive increase in willingness to adopt pro-conservation water usage and behavioral intention to use recycled water, the media messages did not have a significant impact on actual behaviors, such as accepting bottled recycled water or supporting recycled water initiatives. These findings underscored the need for additional interventions beyond web-based videos to effectively promote pro-conservation behaviors.

##### 3.3.5.3 Computer-based concept mapping

Another approach, computer-based concept mapping, involves creating visual representations of an individual’s knowledge structure in a specific domain. Eggert et al. (2017) utilized a concept mapping tool adapted for an experimental manipulation aimed at increasing climate change knowledge. The study divided participants into four experimental conditions centered around climate change themes. Providing students with relevant concepts supported them in generating high-quality concept maps related to climate change conceptual knowledge. However, excessive scaffolding and support had the opposite effect. Students who received argumentative relations were able to visualize their argumentation process regarding climate change solutions. Meanwhile, students in the free mapping condition also demonstrated success in producing high-quality concept maps.

##### 3.3.5.4 Robots

Ham and Midden (2014) researched the persuasive effects of social feedback delivered by a robotic agent. In two experiments, participants were given the task of carrying out washing tasks on a simulated washing machine while having the opportunity to save energy. The social feedback conditions, represented by an iCat–a stylized head of a cat capable of expressing social expressions through movement and speech–were placed on the participants’ desks. Study 1 demonstrated that both social feedback conditions had a significant impact on reducing electricity consumption compared to the control condition, which provided factual feedback about electricity consumption. Negative verbal reactions from a domestic robot were particularly effective in influencing behavior. Study 2 also investigated the influence of task similarity and found that the persuasive effects of negative feedback were enhanced when the tasks were similar.

##### 3.3.5.5 Online tools

Spence et al. (2014) utilized an online tool in a simulation scenario where participants, specifically undergraduates, were presented with an energy display showing their energy use in terms of kilowatt-hour carbon dioxide (CO_2_) emissions, costs, and energy units (kWh). The study found that framing energy reduction in terms of CO_2_ emissions increased the salience of climate change, which, in turn, increased participants’ intentions to engage in environmental behavior. While financial costs may be a more tangible outcome of energy reduction for many individuals, the study emphasized the importance of environmental motivation in driving environmental behavior beyond energy-related actions. In a study by Wheaton et al. (2016), aimed at promoting pro-environmental behaviors (PEBs) after a nature-based tourism intervention, such as a guided tour, it was found that although the intervention had limited overall influence on conservation actions, it was effective in stimulating social media-related actions. Post-tour conservation actions were significantly influenced by participants’ emotional connection to wildlife during the tour and their repeat visitation to either the same or another state park. Additionally, visitors’ connectedness to nature increased during the three-hour tour but returned to pre-visit levels 3 months later, suggesting the need for more sustainable and long-lasting interventions.

### 3.4 RQ 4: sustainable behaviors

**Figure 7:**
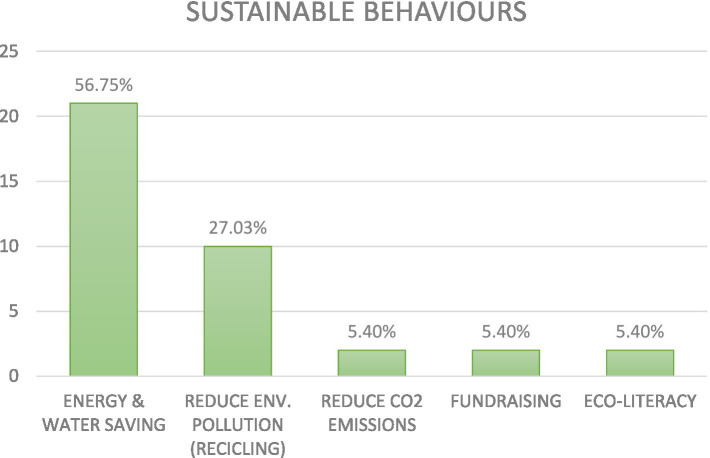
SBs investigated in the screened articles.

Most published articles aimed to promote different types of sustainable behaviors: energy and water saving (56.75%), reducing environmental pollution (27.03%), reducing CO_2_ emissions (5.40%), fund-raising (5.40%) and eco-literacy (5.40%). In the following paragraphs, evidence that emerged for each sustainable behavior is addressed (Figure 7).

#### 3.4.1 Energy and water savings

This category refers to water and energy conservation, or rather, the reduction of such resource waste. To contain the wastefulness of water and energy, the persuasive effect of different types of technologies on these pro-environmental actions was investigated, showing their relevant impact. In particular, the effectiveness of various strategies for promoting pro-environmental behavior includes the combination of a robotic agent and social feedback (Ham and Midden, 2014), the use of vivid and personalized interventions (Bailey et al., 2015), and the utilization of social messages through immersive virtual environment technology (Khashe et al., 2019). Additionally, gamification (Casals et al., 2020; Mahmud et al., 2020), mobile applications (Oliveira et al., 2016), and eco-feedback technologies that provide real-time energy use information (Schwartz et al., 2015; Asensio and Delmas, 2016; Chiu et al., 2020) have been examined in various studies. These interventions have been explored in different contexts, including situations where individuals are not financially responsible for energy costs, such as hotels (Tiefenbeck et al., 2019). However, the long-term impact may not support the maintenance of the positive results achieved during the intervention (Wemyss et al., 2019); indeed, 1 year after using the app, the energy savings were not confirmed.

#### 3.4.2 Reducing environmental pollution

As concerns the actions aimed at the reuse of materials, research has highlighted the complexity of this type of sustainable behavior in changing habits, due to the request of a significant effort from people (von Borgstede and Biel, 2002). Thus, virtual reality has been used to explore its persuasive role in enhancing recycling actions (Chirico et al., 2021), whereas Mozo-Reyes et al. (2016) tested if eco-feedback may influence triggering conservation actions. Results showed that eco-feedback technology increases the reuse activity outside the home. Nevertheless, Schultz and Oskamp (1996) emphasized the cognitive and physical challenges associated with recycling, as individuals must make decisions about what items to recycle.

#### 3.4.3 Reducing CO_2_ emissions

In the context of reducing CO_2_ emissions, transportation plays a significant role in urban air pollution and climate change (Ruscio et al., 2018). Numerous studies have focused on finding effective persuasive strategies to encourage sustainable actions and decrease pollution for the benefit of the environment and human health (Ettema et al., 2016). Technological applications have been identified as valuable tools in promoting eco-friendly activities for reducing CO_2_ emissions (Kimura and Nakajima, 2011).

#### 3.4.4 Eco-literacy

Ecological literacy refers to the knowledge and ability to utilize information related to ecology and sustainability when making decisions. While it is acknowledged that knowledge alone does not guarantee pro-environmental behavior (Skamp et al., 2013), understanding environmental issues is a crucial prerequisite for changing attitudes and intentions to act. Eggert et al. (2017) demonstrated that computer-based concept mapping aids individuals in visualizing and organizing their knowledge, facilitating learning about environmental topics like climate change and aspects of socio-scientific reasoning and decision-making. On the other hand, Schaal et al. (2018) investigated the relationship between geo-games and biodiversity knowledge and found that game-related enjoyment did not predict biodiversity knowledge. However, the study also revealed that game-related enjoyment was a strong predictor of attitudes toward nature.

#### 3.4.5 Fundraising

This type of behavior refers to the act of gathering voluntary contributions such as money or other resources. Non-profit organizations are progressively promoting their causes using technologies (Janpol and Dilts, 2016) and they report increased donations when virtual reality is used. Thus, it appears that technologies can both increase empathy and influence people toward pro-environmental behavior. Nelson et al. (2020) confirmed that virtual reality is an effective way to raise awareness of environmental problems and encourage pro-environmental actions.

## 4 Duration and follow-up

Most published articles did not report the duration of the study (62.50%). Laboratory experiments had a duration of 2 h (Schaal et al., 2018), 90 min sessions repeated four times (Eggert et al., 2017), and 30 min (Harmon and Gauvain, 2019). Studies belonging to the category of power-metering systems reported longer interventions with different durations: 2 months (Tiefenbeck et al., 2019), 18 months (Schwartz et al., 2015), 4 months (Wemyss et al., 2019) 4 years (Henn et al., 2019). Only three studies in the systematic evidence map included a follow-up assessment. One study conducted a follow-up after 8 weeks (Mahmud et al., 2020). Another study (Wemyss et al., 2019) provided results 1 year later, serving as a follow-up to a behavior change intervention named Social Power. This intervention aimed to reduce household electricity consumption by utilizing a gamified mobile application connected to the household’s smart meter. Lastly, in a study by Mahood et al. (2014), a follow-up assessment was conducted 2 weeks after the interventions. In this study, participants in the experimental group received the first message prompting them to take their selected action, followed by up to three additional prompts spaced 1 week apart.

## 5 Population involved

**Figure 8:**
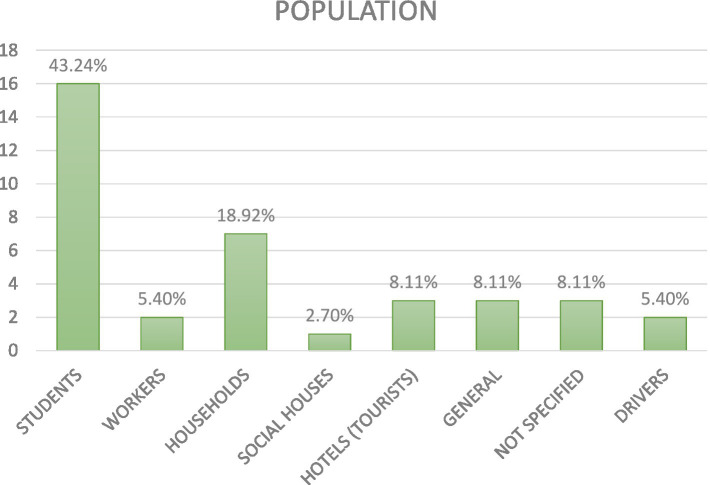
Targets of the interventions in the screened articles.

Regarding the population involved, it was found that over 40% of the studies included in the systematic evidence map focused on students. (43.24%) or civil society in general (8.11%), for experimental studies. Studies involving interventions with power-metering systems targeted inhabitants of social houses (2.70%), inhabitants of different households (18.92%), hotels’ tourists and visitors (8.11%), or workers from offices (5.40%), and only one study employed young drivers (5.40%). In three studies (8.11%) characteristics of the sample were not specified (Figure 8).

## 6 Conclusion

In this systematic mapping, we reviewed 37 articles on digital interventions to determine their effectiveness in promoting SBs outcomes, the type of interventions used, and the type of targeted SBs that have been investigated.

All 37 studies showed at least one positive result (100%); of these, 18 (48.65%) showed only positive outcomes (2, 3, 5, 6, 7, 8, 9, 10, 11, 18, 19, 22, 23, 24, 29, 30, 31, 35), and 19 (51.35%) displayed a mix of positive and negative results (1, 4, 12, 13, 14, 15, 16, 17, 20, 21, 25, 26, 27, 28, 32, 33, 34, 36, 37). Generally, the following factors showed the most successful outcomes: decrease in consumption (37.84%; 1, 2, 3, 6, 7, 8, 14, 19, 24, 28, 29, 31, 33, 35), increase in pro-environmental behaviors/intention to perform pro-environmental behaviors (32.43%; 4, 9, 10, 11, 13, 17, 18, 20, 22, 25, 26, 29), and increase in pro-environmental knowledge/awareness (16.22%; 19, 21, 23, 26, 27, 34).

Of course, publication bias (i.e., primarily positive studies getting published) has to be considered as one of the common confounding factors that could limit the generalizability of this systematic mapping over time. Another issue to be considered is the attitude-behavior gap, with an increasing number of findings confirming this phenomenon substantiated by several methodological flaws (e.g., social desirability bias), contextual (e.g., time pressure and constraints) and/or individual issues (e.g., inability to answer) (Caruana et al., 2016) that influence the existence of such a gap and its magnitude. These issues can be partially overcome by measuring behavior more objectively, for example, by relying on behavioral data, as shown in our section about power-metering systems (e.g., Henn et al., 2019), field experiments with actual behavior as an outcome (e.g., Tiefenbeck et al., 2019). Unobtrusive objective measurement can be applied to several domains: driving behavior can be measured using eco-driving assisting devices coupled with odometers (Ruscio et al., 2018) or GPS devices, energy consumption can be monitored digital meters (Henn et al., 2019) and this can be applied also to water conservation (i.e., smart-show meters), and food waste can be weighed and photographed (Cropley et al., 2022). Enhancing the collection of data on naturally occurring behaviors might increase the chance to collaborate with several non-governative organizations, industrial partners, or public institutions (Lange et al., 2023). Such collaborations have provided results that sometimes are in strong contrast with the ones derived from self-reports (Berger et al., 2022) or that can provide a more strong scientific basis for the behavioral feasibility of technological innovations. Notwithstanding these limitations, due to these positive initial results, it is essential to ensure equity in terms of access to digital tools and at the same time spread digital literacy across all groups in society, in terms of upskilling and reskilling of people. This is particularly needed in today’s era, where digital media has become pervasive, the widespread use of computers, tablets, and smartphones has led to an unprecedented number of internet users (Murthy, 2013). This trend has been further amplified during the recent Covid-19 pandemic, where the necessity of virtual connectedness due to the global crisis has shifted our interactions from face-to-face to technology-based platforms, significantly impacting our lives. The advent of social media networks and digital economies has revolutionized traditional communication channels, reshaping the way we perceive human interaction.

In the realm of digital environmental research, development, and implementation, it is crucial to involve key stakeholders and end users from the early stages and maintain their continuous engagement throughout the design and evaluation of new technologies (Kok et al., 2017). Adopting human-centered design approaches can be instrumental in this process, emphasizing the importance of understanding and empathizing with end users, fostering transdisciplinary collaboration, and incorporating iterative feedback from users to create innovative solutions that are desirable, feasible, and viable (Asbjørnsen et al., 2022). Unfortunately, the reviewed digital interventions did not report best-practice activities and recommendations. On the whole, in contrast to the findings of De Salas et al. (2022) regarding gamified interventions, the studies included in this review offered valuable insights into the technical development practices of these interventions, providing detailed descriptions. Our systematic mapping review revealed the use of diverse data collection tools and measures to report the outcomes of interventions targeting various objectives. While this heterogeneity can be seen as advantageous in addressing the complexity of interventions to promote sustainable behaviors, it limited opportunities for direct comparisons.

In conclusion, our review of these 37 articles investigating pro-environment interventions highlighted that, despite the reported efficacy, the adoption of best-practice intervention designs, as outlined by De Salas et al. (2022) in the context of serious games, was not consistently evident in the studies covered in our mapping. The authors emphasized the importance of understanding existing behaviors, employing best-practice technology development approaches such as multidisciplinary teams and user-centered design, and carefully considering the reasons behind intervention choices. Moreover, the lack of follow-up assessments post-intervention limited the understanding of long-term behavior change, as most studies focused on short-term engagement.

The outcomes of this set of studies do not provide a definitive understanding of which digital interventions are effective and the underlying reasons for their success or failure, indicating a need for further exploration in this field. Consequently, it is premature to offer specific recommendations to policymakers. However, for research funding agencies, we suggest directing resources toward more well-planned, well-executed, and focused studies in this area. To enhance the rigor of future research, several considerations should be addressed. We align with the recommendations of De Salas et al. (2022), advocating for detailed descriptions of intervention design characteristics and the rationale behind design choices, encompassing both behavioral and technological aspects. By doing so, future studies can significantly contribute to the successful adoption of digital interventions in promoting sustainable behavioral changes.

## Author contributions

OM: research ideation, research methodology, design, data collection, data analysis, conceptual model, definitional advancement, and manuscript preparation. AM: data analysis, conceptual model, definitional advancement, and manuscript preparation. SM: data analysis and manuscript preparation. GV: research ideation, and supervision. FF: research ideation, research methodology, design, data collection, data analysis, conceptual model, definitional advancement, manuscript preparation, and supervision. All authors contributed to the article and approved the submitted version.

## Footnotes

^1^The number was initially 54, but one article was excluded because not accessible.

^2^The final number of countries does not correspond to the number of selected studies because in some cases the same study was conducted simultaneously in multiple countries (e.g., Casado-Mansilla et al., 2020).

^3^The total number of sampling techniques used does not correspond to the number of selected studies because in some cases the same study was conducted simultaneously in multiple countries (e.g., Moore and Yang, 2020) or the sample was mixed (e.g., students and the general population; Nelson et al., 2020).
