# Supplementary material for: Digital technologies for behavioral change in sustainability domains: a systematic mapping review
Source: Front Psychol. 2024 Jan 3;14:1234349. doi: 10.3389/fpsyg.2023.1234349 (PMC10795171; doi:10.3389/fpsyg.2023.1234349)
Supplement: Supplementary file 1 [file Table_1.pdf]

Supplementary Table 1. Coding Scheme

DIMENSION	V#	PROPERTY	STATE	TYPE OF VARIABLE
STUDY		ID		Numerical
	V01	YEAR		Numerical
	V02	COUNTRY		Free text
	V03	JOURNAL		Free text
	V04	DESIGN	-Experimental Design:<br>a) Within<br>b) Between<br>c) Mixed methodology<br>- Quasi Experimental Design:<br>a) Within<br>b) Between<br>- Qualitative Longitudinal<br>- Field Experiment	Categories - See states

Supplementary Table 1. Coding Scheme

	V05	SAMPLE STRATEGY	- Convenience sampling:<br>a) Students<br>b) Workers/employees<br>c) Households<br>- Tourists<br>- General population<br>- Crowd-working platforms<br>- Open calls and direct mails	Categories - See states
INTERVENTION	V06	OBJECTIVE		Free text

Supplementary Table 1. Coding Scheme

	V07	TECHNOLOGY TYPE<br>(Independent variable)	- Virtual reality<br>a) 360-degree video features<br>b) Immersive Virtual Environments<br>c) Video games with vividly rendered settings<br>d) Augmented Reality<br>- Energy Management Technologies<br>a) Smart meters<br>b) Smart shower meters<br>c) Eco-feedback technologies<br>- Gamification<br>a) Serious Games<br>b) Geo-games<br>- Eco-driving<br>- Persuasive apps.<br>a) On-line gamified mobile apps<br>- Persuasive robots<br>- Computer-based concept mapping<br>- Videos administered via Internet<br>- On-line tools	Categories - See states

Supplementary Table 1. Coding Scheme

	V07.1	TECHNOLOGY DESCRIPTION		Free text
	V08	SB TYPE<br>(Dependent variable)	- Energy and water saving<br>-Environmental Pollution reduction (e.g., recycling)<br>-Co2 Emissions reduction<br>-Fund-raising<br>-Eco-literacy	Categories - See states
	V08.1	BEHAVIOUR DESCRIPTION		Free text
	V09	COMPARISON		Free text
	V10	MODERATOR		Free text
	V11	MEASURE		Free text
	V12	DURATION		Numerical (time)
	V13	FOLLOW-UP	- Yes<br>- Not	Categories - See states
OUTCOME	V.14	RESULTS		Text/Numerical/Statistics

Supplementary Table 1. Coding Scheme

POPULATION	V15	TARGET	- Students<br>- Workers<br>- Households<br>- Social Houses<br>- Hotels (tourists)<br>- General population<br>- Drivers	Categories - See states
	V16	N.		Numerical
	V17	Experimental Group N.		Numerical
	V18	Control Group N.		Numerical
	V19	AGE		Numerical (mean, range)
	V20	GENDER		Free Text
